# Ventriculocoronary Fistulas with Hypoplastic Left Heart in a Neonate: Imaging with Cardiac CT

**DOI:** 10.1155/2021/6657447

**Published:** 2021-03-16

**Authors:** Serap Baş, Utku Alkara

**Affiliations:** Department of Radiology, İstanbul Yeni Yüzyıl University, Gaziosmanpaşa Hospital, İstanbul, Turkey

## Abstract

Fistulous communications between the ventricular cavities and the coronary arterial tree can be found in the presence of hypoplasia of the left ventricle, especially when the ventricular septum is intact and mitral stenosis and aortic atresia subtype are present. The cardiac CT provides excellent anatomic information especially in the evaluation of extracardiac vessels and coronary arteries. In this case study, we report a newborn with ventriculocoronary fistulas (VCFs) with the hypoplastic left disease diagnosed with cardiac CT. Transthoracic echocardiography of a term baby showed hypoplastic left heart syndrome (HLHS) with mitral stenosis and aortic atresia. The patient immediately underwent a Sano variation of the Norwood procedure. On the postoperative second day, the clinical status of the patient deteriorated. A prospective electrocardiogram-gated axial technique was performed within a single heartbeat for the patient and large VCFs were detected and a second operation were performed to close the VCFs that failed. On the nineteenth day after the operation, the baby passed away. According to us, cardiac CT can also be performed free-breathing and without anesthesia in the neonatal period for the definition of complex cardiac anatomy with the lower radiation dose from the latest scanners, radiation risk of CT should be weighed against the anesthesia risk of cardiac MRI and intraoperative risk of conventional cardiac angiography. Pre-operative cardiac CT may increase surgical success.

## 1. Introduction

Hypoplastic left heart syndrome (HLHS) represents 2% to 9% of congenital heart disease cases and accounts for 23% of neonatal deaths from congenital heart malformations [1]. Fistulous communications between the ventricular cavities and the coronary arterial tree can be found in the presence of hypoplasia of the left ventricle, especially when the ventricular septum is intact and mitral stenosis and aortic atresia subtype are present [2]. Various imaging modalities play a role in the diagnosis of congenital heart diseases, such as echocardiography, cardiac MRI, conventional angiography, and cardiac computer tomography (CT) angiography. Cardiac CT angiography is a noninvasive tool with high image spatial resolution and powerful 3D dimensional postprocessing image reconstruction. This provides excellent anatomic information that can work together with and can also replace echocardiography and cardiac catheterization, especially in the evaluation of extracardiac vessels and coronary arteries [3].

In this case study, we report a newborn with ventriculocoronary fistulas with the hypoplastic left disease diagnosed with cardiac CT.

## 2. Case Report

**Figure 1:**
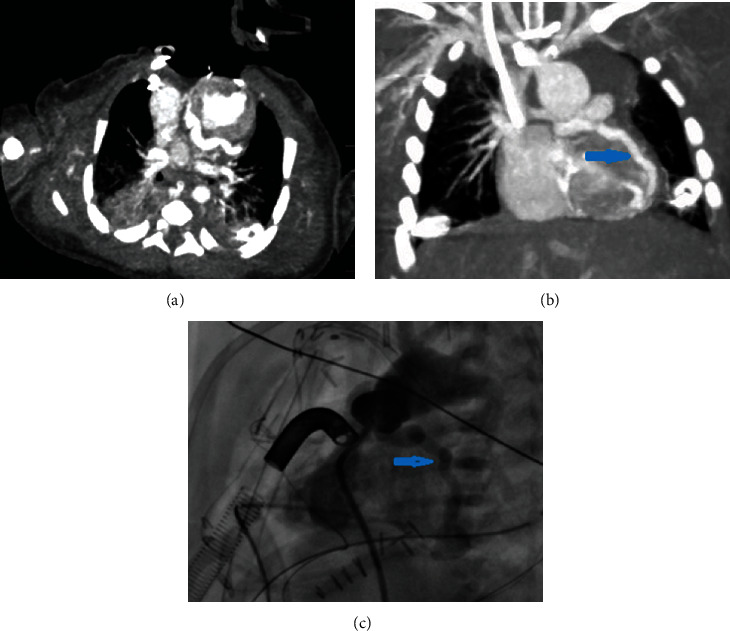
(a) Axial image of dilated left anterior descending artery. (b) Coronal image of ventriculocoronary fistulas between the left anterior descending artery and the left ventricle (blue arrow). (c) Conventional angiography of ventriculocoronary fistulas (blue arrow).

A term baby was admitted to the neonatal intensive care unit following a cesarian delivery because of hypoxemia and respiratory distress. Transthoracic echocardiography showed HLHS with mitral stenosis and aortic atresia. There was no additional knowledge about coronary circulation. The patient immediately underwent a Sano variation of the Norwood procedure. During the operation, left anterior descending (LAD) was seen tortuous and aneurysmatic. The patient was stable during the early postoperative period. On the postoperative second day, the clinical status of the patient deteriorated, and electrocardiography exhibited sinus tachycardia with (ST) elevation in the left precordial leads. Electrocardiogram-gated cardiac CT was administered, and large ventriculocoronary fistulas were detected. Conventional angiography and a second operation were performed to close the ventriculocoronary fistulas that did not succeed. On the nineteenth day after the operation, the baby passed away due to heart failure (Figure 1).

Cardiac CT was performed on a single source 512 slices CT (Revolution CT, General Electric Healthcare, Milwaukee) using a wide detector aperture (160 mm) iterative reconstruction algorithm (ASIR-V with 50% strength) and specific reconstruction software reducing coronary motion artifacts (snapshot freeze). A prospective electrocardiogram-gated axial technique was performed within a single heartbeat for the patient. A low KV value was selected to maximize the iodine contrast to noise ratio (70 kV). Iodinated contrast medium (Omnipaque 350 mg/ml) at 2 ml per kg was intravenously administered, followed by intravenously 10 ml of saline solution. Contrast material was infused with a flow rate of 1.5 ml/s through an intravenous catheter. The scan volume was set to the whole chest, extending from the supraclavicular level superiorly to just below the diaphragm inferiorly. The center for the data acquisition phase window was set to 45% of the R-R interval due to the heart rate of the patient, which was over 160 bpm. The consent was obtained from the patients' parents for this case report.

## 3. Discussion

Ventriculocoronary fistulas, also known as sinusoids, were reported in the hypoplastic left heart. Overall, VCFs were found in 15% of the hypoplastic left heart cases. They occur in 56% of the mitral stenosis and aortic atresia subtype [2]. Vida et al. found a 50% failure rate for stage 1 palliation in patients with this anatomic variant, and Sathanandam et al. suggested that large VCFs were associated with poor prognosis [2, 4]. Some authors have proposed that VCFs might interfere with adequate myocardial protection during heart decompression on cardiopulmonary bypass, resulting in ischemia-induced ventricular dysfunction [4, 5]. Matsushima et al. presented a neonate with known HLHS and VCFs who underwent a successful, modified, beating-heart Norwood operation with coronary perfusion [6].

Comprehensive anatomic evaluation in complex congenital heart disease is critical to effective patient management. In the very young pediatric population, coronary artery diameters are smaller, and heart rates are higher. When planning surgical intervention, the detection of coronary anomalies, which are frequently associated with complex congenital heart disease, is a major significance to avoid fatal complications during surgery due to incorrect anatomic information.

Echocardiography is often used to diagnose congenital heart defects (CHD), but the limits of the acoustic window, poor spatial resolution, and the subjectivity of the operator's judgments are major drawbacks to this procedure. Sometimes, coronary arteries are too small to visualize and cannot be detected by echocardiography [3].

Neonatal cardiac MRI involves relatively long imaging times, requires general anesthesia with intubation for suspended respiration, and decreases signal-to-noise ratios, and high baseline heart rates pose technical challenges in the smallest patients. Because of the long imaging times, MRI is not an appropriate imaging modality of choice for critically ill patients [3].

Conventional cardiac angiography is an invasive procedure with an approximately 1% intraoperative mortality rate. Catheterization of an infant or a young child, especially one with complex CHD, is rather difficult because of the patient's small size and inability to cooperate. Many neonates with complex CHD are the most likely to have hemodynamic effects from changes in systemic or pulmonary pressures as a result of anesthesia or suspended respiration. Repeated or prolonged use of general anesthetics or sedative drugs during childhood may be associated with negative effects on the developing brain [7].

Cardiac CT has been progressively used as a diagnostic tool complementary to echocardiography. Cardiac CT provides superior spatial resolution with multiple reconstruction possibilities. It is also a noninvasive and widely available technique. The recent development of a wide detector scanner allows volumetric acquisitions within a single heartbeat, avoiding stair-step artifacts, reducing radiation exposure, and improving uniform contrast enhancement.

## 4. Conclusion

According to us, cardiac CT can also be performed free-breathing and without anesthesia in the neonatal period for the definition of complex cardiac anatomy with the lower radiation dose from the latest scanners; radiation risk of CT should be weighed against the anesthesia risk of cardiac MRI and intraoperative risk of conventional cardiac angiography.
